# Characteristics, management, and prognosis of elderly patients with COVID-19 admitted in the ICU during the first wave: insights from the COVID-ICU study: Prognosis of COVID-19 elderly critically ill patients in the ICU

**DOI:** 10.1186/s13613-021-00861-1

**Published:** 2021-05-14

**Authors:** Martin Dres, David Hajage, Said Lebbah, Antoine Kimmoun, Tai Pham, Gaëtan Béduneau, Alain Combes, Alain Mercat, Bertrand Guidet, Alexandre Demoule, Matthieu Schmidt, the COVID-ICU investigators, Alain Mercat, Pierre Asfar, François Beloncle, Julien Demiselle, Tài Pham, Arthur Pavot, Xavier Monnet, Christian Richard, Alexandre Demoule, Martin Dres, Julien Mayaux, Alexandra Beurton, Cédric Daubin, Richard Descamps, Aurélie Joret, Damien Du Cheyron, Frédéric Pene, Jean-Daniel Chiche, Mathieu Jozwiak, Paul Jaubert, Guillaume Voiriot, Muriel Fartoukh, Marion Teulier, Clarisse Blayau, Erwen L’Her, Cécile Aubron, Laetitia Bodenes, Nicolas Ferriere, Johann Auchabie, Anthony Le Meur, Sylvain Pignal, Thierry Mazzoni, Jean-Pierre Quenot, Pascal Andreu, Jean-Baptiste Roudau, Marie Labruyère, Saad Nseir, Sébastien Preau, Julien Poissy, Daniel Mathieu, Sarah Benhamida, Rémi Paulet, Nicolas Roucaud, Martial Thyrault, Florence Daviet, Sami Hraiech, Gabriel Parzy, Aude Sylvestre, Sébastien Jochmans, Anne-Laure Bouilland, Mehran Monchi, Marc Danguy des Déserts, Quentin Mathais, Gwendoline Rager, Pierre Pasquier, Reignier Jean, Seguin Amélie, Garret Charlotte, Canet Emmanuel, Jean Dellamonica, Clément Saccheri, Romain Lombardi, Yanis Kouchit, Sophie Jacquier, Armelle Mathonnet, Mai-AhnNay, Isabelle Runge, Frédéric Martino, Laure Flurin, Amélie Rolle, Michel Carles, Rémi Coudroy, Arnaud W. Thille, Jean-Pierre Frat, Maeva Rodriguez, Pascal Beuret, Audrey Tientcheu, Arthur Vincent, Florian Michelin, Marie Anne Melone, Maxime Gauzi, Arnaud Guilbert, Geoffrey Kouadri, Valérie Gissot, Stéphan Ehrmann, Charlotte Salmon Gandonniere, Djlali Elaroussi, Agathe Delbove, Yannick Fedun, Julien Huntzinger, Eddy Lebas, Grâce Kisoka, Céline Grégoire, Stella Marchetta, Bernard Lambermont, Laurent Argaud, Thomas Baudry, Pierre-Jean Bertrand, Auguste Dargent, Christophe Guitton, Nicolas Chudeau, Mickaël Landais, Cédric Darreau, Alexis Ferre, Antoine Gros, Guillaume Lacave, Fabrice Bruneel, Mathilde Neuville, Jérôme Devaquet, Guillaume Tachon, Richard Gallot, Riad Chelha, Arnaud Galbois, Anne Jallot, Ludivine Chalumeau Lemoine, Khaldoun Kuteifan, Valentin Pointurier, Louise-Marie Jandeaux, Joy Mootien, Charles Damoisel, Benjamin Sztrymf, Matthieu Schmidt, Alain Combes, Juliette Chommeloux, Charles Edouard Luyt, Frédérique Schortgen, Leon Rusel, Camille Jung, Florent Gobert, Damien Vimpere, Lionel Lamhaut, Bertrand Sauneuf, Liliane Charrrier, Julien Calus, Isabelle Desmeules, Benoît Painvin, Jean-Marc Tadie, Vincent Castelain, Baptiste Michard, Jean-Etienne Herbrecht, Mathieu Baldacini, Nicolas Weiss, Sophie Demeret, Clémence Marois, Benjamin Rohaut, Pierre-Henri Moury, Anne-Charlotte Savida, Emmanuel Couadau, Mathieu Série, Nica Alexandru, Cédric Bruel, Candice Fontaine, Sonia Garrigou, Juliette Courtiade Mahler, Maxime Leclerc, Michel Ramakers, Pierre Garçon, Nicole Massou, Ly Van Vong, Juliane Sen, Nolwenn Lucas, Franck Chemouni, Annabelle Stoclin, Alexandre Avenel, Henri Faure, Angélie Gentilhomme, Sylvie Ricome, Paul Abraham, Céline Monard, Julien Textoris, Thomas Rimmele, Florent Montini, Gabriel Lejour, Thierry Lazard, Isabelle Etienney, Younes Kerroumi, Claire Dupuis, Marine Bereiziat, Elisabeth Coupez, François Thouy, Clémet Hoffmann, Nicolas Donat, Violaine Muller, Thibault Martinez, Antoine Kimmoun, Audrey Jacquot, Matthieu Mattei, Bruno Levy, Ramin Ravan, Loïc Dopeux, Jean-Mathias Liteaudon, Delphine Roux, Brice Rey, Radu Anghel, Deborah Schenesse, Vincent Gevrey, Jermy Castanera, Philippe Petua, Benjamin Madeux, Otto Hartman, Michael Piagnerelli, Anne Joosten, Cinderella Noel, Patrick Biston, Thibaut Noel, Gurvan L. E. Bouar, Messabi Boukhanza, Elsa Demarest, Marie-France Bajolet, Nathanaël Charrier, Audrey Quenet, Cécile Zylberfajn, Nicolas Dufour, Buno Mégarbane, SqébastianVoicu, Nicolas Deye, Isabelle Malissin, François Legay, Matthieu Debarre, Nicolas Barbarot, Pierre Fillatre, Bertrand Delord, Thomas Laterrade, Tahar Saghi, Wilfried Pujol, Pierre Julien Cungi, Pierre Esnault, Mickael Cardinale, Vivien Hong Tuan Ha, Grégory Fleury, Marie-Ange Brou, Daniel Zafimahazo, David Tran-Van, Patrick Avargues, Lisa Carenco, Nicolas Robin, Alexandre Ouali, Lucie Houdou, Christophe Le Terrier, Noémie Suh, Steve Primmaz, Jérome Pugin, Emmanuel Weiss, Tobias Gauss, Jean-Denis Moyer, Catherine Paugam Burtz, Béatrice La Combe, Rolland Smonig, Jade Violleau, Pauline Cailliez, Jonathan Chelly, Antoine Marchalot, Cécile Saladin, Christelle Bigot, Pierre-Marie Fayolle, Jules Fatséas, Amr Ibrahim, Dabor Resiere, Rabih Hage, Clémentine Cholet, Marie Cantier, Pierre Trouiler, Philippe Montravers, Brice Lortat-Jacob, Sebastien Tanaka, Alexy Tran Dinh, Jacques Duranteau, Anatole Harrois, Guillaume Dubreuil, Marie Werner, Anne Godier, Sophie Hamada, Diane Zlotnik, Hélène Nougue, Armand Mekontso-Dessap, Guillaume Carteaux, Keyvan Razazi, Nicolas De Prost, Nicolas Mongardon, Olivier Langeron, Eric Levesque, Arié Attias, Charles de Roquetaillade, Benjamin G. Chousterman, Alexandre Mebazaa, Etienne Gayat, Marc Garnier, Emmanuel Pardo, Lea Satre-Buisson, Christophe Gutton, Elise Yvin, Clémence Marcault, Elie Azoulay, Michael Darmon, Hafid Ait Oufella, Geoffroy Hariri, Tomas Urbina, Sandie Mazerand, Nicholas Heming, Francesca Santi, Pierre Moine, Djillali Annane, Adrien Bouglé, Edris Omar, Aymeric Lancelot, Emmanuelle Begot, Gaétan Plantefeve, Damien Contou, Hervé Mentec, Olivier Pajot, Stanislas Faguer, Olivier Cointault, Laurence Lavayssiere, Marie-Béatrice Nogier, Matthieu Jamme, Claire Pichereau, Jan Hayon, Hervé Outin, François Dépret, Maxime Coutrot, Maité Chaussard, Lucie Guillemet, Pierre Goffin, Romain Thouny, Julien Guntz, Laurent Jadot, Romain Persichini, Vanessa Jean-Michel, Hugues Georges, Thomas Caulier, Gaël Pradel, Marie-Hélène Hausermann, ThiMy Hue Nguyen-Valat, Michel Boudinaud, Emmanuel Vivier, Sylvène Rosseli, Gaël Bourdin, Christian Pommier, Marc Vinclair, Simon Poignant, Sandrine Mons, Wulfran Bougouin, Franklin Bruna, Quentin Maestraggi, Christian Roth, Laurent Bitker, François Dhelft, Justine Bonnet-Chateau, Mathilde Filippelli, Tristan Morichau-Beauchant, Stéphane Thierry, Charlotte Le Roy, Mélanie Saint Jouan, Bruno Goncalves, Aurélien Mazeraud, Matthieu Daniel, Tarek Sharshar, Cyril Cadoz, RostaneGaci, Sébastien Gette, Guillaune Louis, Sophe-Caroline Sacleux, Marie-Amélie Ordan, Aurélie Cravoisy, Marie Conrad, Guilhem Courte, Sébastien Gibot, Younès Benzidi, Claudia Casella, Laurent Serpin, Jean-Lou Setti, Marie-Catherine Besse, Anna Bourreau, Jérôme Pillot, Caroline Rivera, Camille Vinclair, Marie-Aline Robaux, Chloé Achino, Marie-Charlotte Delignette, Tessa Mazard, Frédéric Aubrun, Bruno Bouchet, Aurélien Frérou, Laura Muller, Charlotte Quentin, Samuel Degoul, Xavier Stihle, Claude Sumian, Nicoletta Bergero, Bernard Lanaspre, Hervé Quintard, Eve Marie Maiziere, Pierre-Yves Egreteau, Guillaume Leloup, Florin Berteau, Marjolaine Cottrel, Marie Bouteloup, Matthieu Jeannot, Quentin Blanc, Julien Saison, Isabelle Geneau, Romaric Grenot, Abdel Ouchike, Pascal Hazera, Anne-Lyse Masse, Suela Demiri, Corinne Vezinet, Elodie Baron, Deborah Benchetrit, Antoine Monsel, Grégoire Trebbia, Emmanuelle Schaack, Raphaël Lepecq, Mathieu Bobet, Christophe Vinsonneau, Thibault Dekeyser, Quentin Delforge, Imen Rahmani, Bérengère Vivet, Jonathan Paillot, Lucie Hierle, Claire Chaignat, Sarah Valette, Benoït Her, Jennifier Brunet, Mathieu Page, Fabienne Boiste, Anthony Collin, Florent Bavozet, Aude Garin, Mohamed Dlala, Kais Mhamdi, Bassem Beilouny, Alexandra Lavalard, Severine Perez, Benoit Veber, Pierre-Gildas Guitard, Philippe Gouin, Anna Lamacz, Fabienne Plouvier, Bertrand P. Delaborde, Aïssa Kherchache, Amina Chaalal, Jean-Damien Ricard, Marc Amouretti, Santiago Freita-Ramos, Damien Roux, Jean-Michel Constantin, Mona Assefi, Marine Lecore, Agathe Selves, Florian Prevost, Christian Lamer, Ruiying Shi, Lyes Knani, Sébastien PiliFloury, Lucie Vettoretti, Michael Levy, Lucile Marsac, Stéphane Dauger, Sophie Guilmin-Crépon, Hadrien Winiszewski, Gael Piton, Thibaud Soumagne, Gilles Capellier, Jean-Baptiste Putegnat, Frédérique Bayle, Maya Perrou, Ghyslaine Thao, Guillaume Géri, Cyril Charron, Xavier Repessé, Antoine Vieillard-Baron, Mathieu Guilbart, Pierre-Alexandre Roger, Sébastien Hinard, Pierre-Yves Macq, Kevin Chaulier, Sylvie Goutte, Patrick Chillet, Anaïs Pitta, Barbara Darjent, Amandine Bruneau, Sigismond Lasocki, Maxime Leger, Soizic Gergaud, Pierre Lemarie, Nicolas Terzi, Carole Schwebel, Anaïs Dartevel, Louis-Marie Galerneau, Jean-Luc Diehl, Caroline Hauw-Berlemont, Nicolas Péron, Emmanuel Guérot, Abolfazl Mohebbi Amoli, Michel Benhamou, Jean-Pierre Deyme, Olivier Andremont, Diane Lena, Julien Cady, Arnaud Causeret, Arnaud De La Chapelle, Christophe Cracco, Stéphane Rouleau, David Schnell, Camille Foucault, Cécile Lory, Thibault Chapelle, Vincent Bruckert, Julie Garcia, Abdlazize Sahraoui, Nathalie Abbosh, Caroline Bornstain, Pierre Pernet, Florent Poirson, Ahmed Pasem, Philippe Karoubi, Virginie Poupinel, Caroline Gauthier, François Bouniol, Philippe Feuchere, Florent Bavozet, Anne Heron, Serge Carreira, Malo Emery, Anne Sophie Le Floch, Luana Giovannangeli, Nicolas Herzog, Christophe Giacardi, Thibaut Baudic, Chloé Thill, Said Lebbah, Jessica Palmyre, Florence Tubach, David Hajage, Nicolas Bonnet, Nathan Ebstein, Stéphane Gaudry, Yves Cohen, Julie Noublanche, Olivier Lesieur, Arnaud Sément, Isabel Roca-Cerezo, Michel Pascal, Nesrine Sma, Gwenhaël Colin, Jean-Claude Lacherade, Gauthier Bionz, Natacha Maquigneau, Pierre Bouzat, Michel Durand, Marie-Christine Hérault, Jean-Francois Payen

**Affiliations:** 1 Médecine Intensive Réanimation (Département R3S), Service de Médecine intensive Réanimation, Groupe Hospitalier Universitaire APHP-Sorbonne Université, Hôpital de la Pitié–Salpêtrière, Site Pitié-Salpêtrière, 47-73, bd de l’Hôpital, 75651 Paris Cedex 13, France; 2 UMR S 1136, Sorbonne Université INSERM UMRS_1158 Neurophysiologie Respiratoire Expérimentale et Clinique, Paris, France; 3 Sorbonne Université, INSERM, Institut Pierre Louis d’Epidémiologie et de Santé Publique, Paris, France; 4 Unité de Recherche Clinique, AP-HP, Paris, France; 5 Service de Médecine Intensive et Réanimation Brabois, Université de Lorraine, CHRU de Nancy, Paris, France; 6 INSERM U1116, Vandoeuvre-les-Nancy, France; 7 Service de Médecine Intensive-Réanimation, Hôpital Bicêtre, Hôpitaux Universitaires Paris-Saclay, Le Kremlin-Bicêtre, France; 8 Équipe d’Épidémiologie Respiratoire Intégrative, Center for Epidemiology and Population Health (CESP), Université Paris-Saclay, UVSQ, Univ. Paris-Sud, Inserm, Villejuif, France; 9 Normandie Univ, UNIROUEN, EA 3830, Rouen, France; 10 Medical Intensive Care Unit, Rouen University Hospital, 76000 Rouen, France; 11 Sorbonne Université, INSERM, UMRS_1166-iCAN, Institute of Cardiometabolism and Nutrition, 75651 Paris Cedex 13, France; 12 Médecine intensive Réanimation, Assistance Publique-Hôpitaux de Paris, Pitié-Salpêtrière Hospital, 75651 Paris Cedex 13, France; 13 Département de Médecine Intensive-Réanimation et Médecine Hyperbare, Faculté de Santé, CHU d’Angers, Université d’Angers, Angers, France; 14 Médecine intensive Réanimation, APHP-Sorbonne Université, Hôpital Saint Antoine, Paris, France

**Keywords:** Acute respiratory distress syndrome, Intubation, COVID-19, Mortality, Old patients, Intensive care unit, Frailty

## Abstract

**Background:**

The COVID-19 pandemic is a heavy burden in terms of health care resources. Future decision-making policies require consistent data on the management and prognosis of the older patients (> 70 years old) with COVID-19 admitted in the intensive care unit (ICU).

**Methods:**

Characteristics, management, and prognosis of critically ill old patients (> 70 years) were extracted from the international prospective COVID-ICU database. A propensity score weighted-comparison evaluated the impact of intubation upon admission on Day-90 mortality.

**Results:**

The analysis included 1199 (28% of the COVID-ICU cohort) patients (median [interquartile] age 74 [72–78] years). Fifty-three percent, 31%, and 16% were 70–74, 75–79, and over 80 years old, respectively. The most frequent comorbidities were chronic hypertension (62%), diabetes (30%), and chronic respiratory disease (25%). Median Clinical Frailty Scale was 3 (2–3). Upon admission, the PaO_2_/FiO_2_ ratio was 154 (105–222). 740 (62%) patients were intubated on Day-1 and eventually 938 (78%) during their ICU stay. Overall Day-90 mortality was 46% and reached 67% among the 193 patients over 80 years old. Mortality was higher in older patients, diabetics, and those with a lower PaO_2_/FiO_2_ ratio upon admission, cardiovascular dysfunction, and a shorter time between first symptoms and ICU admission. In propensity analysis, early intubation at ICU admission was associated with a significantly higher Day-90 mortality (42% vs 28%; hazard ratio 1.68; 95% CI 1.24–2.27; *p* < 0·001).

**Conclusion:**

Patients over 70 years old represented more than a quarter of the COVID-19 population admitted in the participating ICUs during the first wave. Day-90 mortality was 46%, with dismal outcomes reported for patients older than 80 years or those intubated upon ICU admission.

**Supplementary Information:**

The online version contains supplementary material available at 10.1186/s13613-021-00861-1.

## Introduction

The severe acute respiratory syndrome coronavirus 2 (SARS-CoV-2) is a risk factor for acute respiratory distress syndrome (ARDS) that is currently a major healthcare challenge worldwide. The prognosis of this disease widely varies between countries, the age of the patients, the characteristics of the population studied, and the severity of the ARDS [1]. Then, the case fatality rates observed in ARDS-related SARS-CoV-2 is close to 30–40% [2–4], but can reach 70% in the older patients [5–7]. Given the heavy burden of ARDS-related SARS-CoV-2 infection in terms of health care resources and the worrisome prognosis of this disease, the pandemic has raised several ethical questions. One of them is the decision to admit the oldest patients in the ICU [8], which should be guided by robust data on the outcomes of that population. Therefore, there is an urgent need to provide consistent data on the management and prognosis of the elderly patients in the intensive care unit (ICU) [9]. These data may serve policymakers to properly and fairly allocate health care resources to that population and also to provide transparent information to the patient and caregivers. To date, few studies specifically reported the management and prognosis of the elderly patients in the context of SARS-CoV-2 lower respiratory tract infection [10, 11], but none were focused on a population admitted in ICU. In a large German study enrolling 10,021 patients, 923 (9%) patients over 70 years old received ventilatory support which was associated with 63% in-hospital mortality in those 70–79 years [4]. This result concurred with the dismal prognosis reported in previous studies focused on elderly patients with ARDS not related to SARS-CoV-2 infection [12, 13]. As the debate is still active whether the management of COVID-19 should differ from ARDS related to other causes [14], the specific ICU management and outcomes of the old patients with SARS-CoV-2 related ARDS has not been fully described so far. We sought to assess the characteristics, management, and prognosis of the patients over 70 years enrolled in the international COVID-ICU cohort [15].

## Methods

### Study design, patients

We performed an ancillary analysis of the COVID-ICU study. COVID-ICU was a multi-center, observational, and prospective cohort study conducted in 149 ICUs from 138 centers, across three countries (France, Switzerland, and Belgium) and has been described elsewhere [15]. It received approval from the ethical committee of the French Intensive Care Society (CE-SRLF 20-23) and Swiss and Belgium ethical committees following local regulations. All patients or close relatives were informed that their medical data were anonymously included in the COVID-ICU cohort. Patients and relatives had the possibility not to participate in the study. In case of refusal, the data were not collected accordingly. This manuscript follows the STROBE statement for reporting cohort studies.

For this analysis, we restricted the study population to patients who were 70 and above 70 years of age at the time of the admission to the participating ICU between February 25, 2020, and May 4, 2020, with laboratory-confirmed SARS-CoV-2 infection, and available Day-90 vital status. Laboratory confirmation for SARS-Cov-2 was defined as a positive result of real-time reverse transcriptase-polymerase chain reaction (RT-PCR) assay from either nasal or pharyngeal swabs, or lower respiratory tract aspirates [16].

### Data collection

Full description of data collection is provided in the Additional file 1. Baseline information collected at ICU admission were: age, sex, body mass index (BMI), active smoking, Simplified Acute Physiology Score (SAPS) II score [17], worse Sequential Organ Failure Assessment (SOFA) [18] during the first 24 h, comorbidities, immunodeficiency (if present), Clinical Frailty Scale [19], the date of the first symptom, and dates of the hospital and ICU admissions. The Clinical Frailty Scale was collected upon ICU admission by the physician in charge of the patient during the medical examination. If the patient was not able to communicate, the physician obtained the information from the relatives. The Clinical Frailty Scale is an ordinal hierarchical scale of 9 ranks, with a score of 1 being very fit, 2 well, 3 managing well, 4 vulnerable, 5 mildly frail, 6 moderately frail, 7 severely frail, 8 very severely frail, and 9 terminally ill. We also collected modes of ventilation and oxygenation and complications over the ICU stay. Patient outcomes included duration of mechanical ventilation, vital status at ICU and hospital discharge, and 28, 60, and 90 days after ICU admission. Lastly, life-sustaining treatment decisions were also collected.

### Statistical analyses

Characteristics of patients were described as frequencies and percentages for categorical variables, whereas continuous variables were reported as mean and standard deviation or median and interquartile range. Categorical variables were compared by Chi-square or Fisher's exact test, and continuous variables were compared by Student's *t*-test or Wilcoxon's rank-sum test. Kaplan–Meier overall survival curves until Day-90 were computed, and were compared using log-rank tests. Detailed statistical analysis is provided is the Additional file 1.

Baseline risk factors of death at Day-90 were assessed using univariate and multivariate Cox regression model stratified on the center variable. Proportional hazard assumption was assessed by inspecting the scaled Schoenfeld residuals and Harrell’s test [20]. To assess invasive mechanical ventilation effect on Day-90 mortality, we used a Cox proportional hazard model weighted on inverse probability of treatment weighting (IPTW) using propensity score (PS) defined as the predictive probability of invasive mechanical ventilation conditional on measured baseline covariates [21]. A multivariate logistic regression model was performed to estimate the PS for each patient in that population. To assess the balance of measured covariates between treatment groups, we used the standardized mean differences before and after PS weighting [22]. Then, a Cox proportional hazard model weighted on IPTW was performed to estimate the average treatment effect in the entire eligible population [21]. Hazard ratio and its 95% confidence interval were then estimated for the Day-90 mortality associated with invasive mechanical ventilation at Day-1. This analysis was performed on the complete cases data set, and a sensitivity analysis was performed using multiple imputations due to missing data.

All analyses were performed at a two-sided α level of 5% and conducted with R version 3.5.1 (R Foundation for Statistical Computing, Vienna, Austria).

## Results

### Characteristics of patients at ICU admission

From the 4244 patients enrolled in the COVID-ICU dataset, 1199 (28%) (1115, 41, 43 patients in France, Switzerland, and Belgium, respectively) met the inclusion criteria of the present study (i.e., age over 70 years old) (see the Additional file 1: Figure S1). The main descriptors of the patient’s characteristics are presented in Table 1. The median (IQR) age was 74 (72–78) years. Fifty-three percent of the patients were 70–74 years old, 31% were 75–79 years old and 16% were over 80 years old. The majority of the patients were male (73%). The most frequent comorbidities were chronic hypertension (62%), diabetes (30%), and chronic respiratory disease (25%). Noticeably, the median (IQR) Clinical Frailty Scale was 3 (2–3), with only 160/1085 (15%) vulnerable patients (i.e., Clinical Frailty Scale 4), and 99/1085 (9%) frail patients (i.e., Clinical Frailty Scale 5–9). The time between first symptoms and ICU admission was 8 (6–12) days. SAPS II and SOFA scores at ICU admission were 43 (35–54) and 5 (3–8), respectively.

**Table 1:** Demographic characteristics and management during the first 14 days of ICU according to their Day-90 survival status

	All patients *n*=1199	Day-90 status		*P* value
		Alive *n*=650	Death *n*=549	
Age, years	74 (72–78)	73 (71–77)	75 (72–79)	< 0.001
70–74	639 (53)	392 (60)	247 (45)	
75–79	367 (31)	194 (30)	173 (32)	
> 80	193 (16)	64 (9)	129 (24)	
Body mass index, kg m^–2^	27 (25–31)	27 (25–31)	27 (25–30)	0.452
Female gender	326 (27)	177 (27)	149 (27)	0.989
Living place				0.007
Home residency	1136 (95)	624 (96)	512 (94)	
Rehabilitation	14 (1)	5 (1)	9 (2)	
Retirement home	20 (2)	4 (1)	16 (3)	
Other	29 (2)	17 (2)	12 (2)	
Comorbidities				
Hypertension	742 (62)	399 (62)	343 (63)	0.728
Diabetes	355 (30)	160 (25)	195 (36)	< 0.001
Active smokers	46 (4)	21 (3)	25 (5)	0.201
Chronic respiratory disease	297 (25)	156 (24)	141 (26)	0.472
Chronic cardiac disease	87 (8)	34 (5)	53 (10)	0.003
Chronic renal insufficiency	108 (9)	44 (7)	64 (12)	0.003
Immunosuppression	99 (8)	45 (7)	54 (10)	0.062
Clinical Frailty Scale	3 (2–3)	3 (2–3)	3 (2–4)	< 0.001
1–3	826 (76)	498 (85)	328 (66)	
4	160 (15)	62 (11)	98 (20)	
5–9	99 (9)	29 (5)	70 (14)	
ICU admission				
Time between hospital and ICU admission, days	1 (0–3)	1 (0–3)	0 (0–2)	0.066
Time between first signs and ICU admission, days	8 (6–12)	10 (6–13)	7 (5–10)	< 0.001
SAPS II	43 (35–54)	41 (33–51)	47 (38–57)	< 0.001
SOFA score	5 (3–8)	4 (3–8)	6 (4–9)	< 0.001
Renal component	0 (0–1)	0 (0–0)	0 (0–1)	< 0.001
Cardiovascular component	1 (0–4)	0 (0–3)	3 (0–4)	< 0.001
During the first 24 hours in the ICU				
PaO_2_/FiO_2_ ratio	154 (105–222)	167 (115–224)	139 (94–212)	0.004
Standard oxygen	339 (29)	210 (33)	129 (24)	< 0.001
Flow, L/min	9 (6–15)	7 (5–15)	12 (7–15)	< 0.001
High-flow oxygen therapy	249 (21)	150 (24)	99 (18)	0.025
Flow, L/min	50 (40–60)	50 (40–60)	50 (40–50)	0.295
FiO_2_, %	75 (60–94)	70 (60–85)	90 (70–100)	< 0.001
Invasive mechanical ventilation	740 (62)	350 (54)	390 (71)	< 0.001
Prone positioning	146 (20)	61 (18)	85 (22)	0.172
Continuous neuromuscular blockades	517 (43)	251 (39)	266 (48)	0.383
During the first 14 days in the ICU				
High-flow oxygen therapy	331 (28)	208 (32)	123 (23)	0.002
Invasive mechanical ventilation	936 (78)	461 (71)	475 (87)	< 0.001
Prone positioning	613 (51)	274 (42)	339 (62)	0.001
Continuous neuromuscular blockades	803 (67)	390 (60)	413 (75)	0.165
Renal replacement therapy	231 (19)	84 (13)	147 (26)	< 0.001
Corticosteroids	409 (34)	191 (30)	218 (40)	< 0.001
Life sustaining treatment decision	253 (21)	30 (5)	223 (41)	< 0.001

Values are expressed as median (interquartile range) or *n* (%)

*ICU* intensive care unit, *SAPS* simplified acute physiology score, *SOFA* Sequential Organ Failure Assessment

**Fig. 1:**
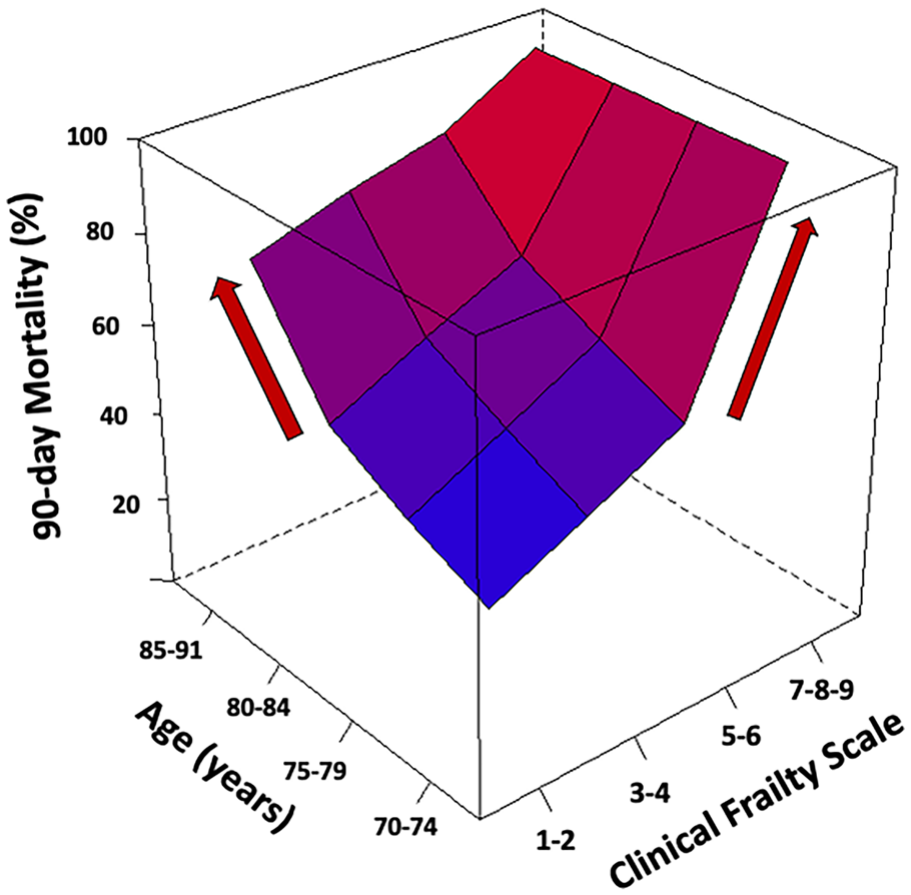
Day-90 mortality according to age and Clinical Frailty Scale

**Fig. 2:**
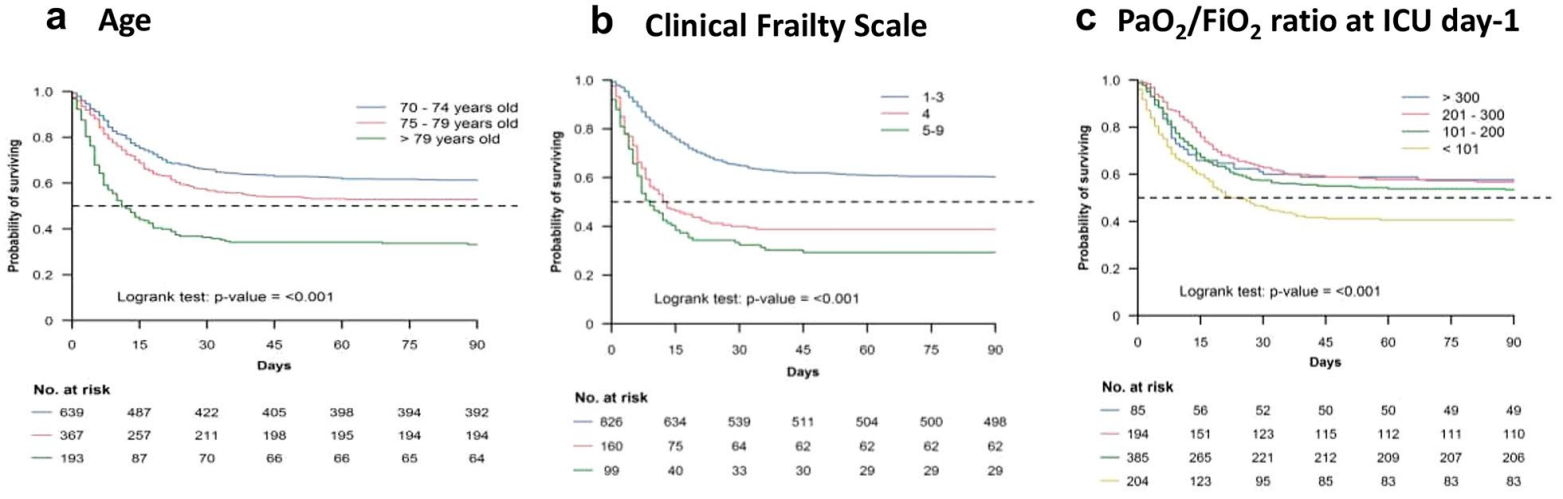
Kaplan–Meier survival estimates during the 90 days following ICU admission, according to **a** age (70–74 years, 75–79 years and > 80 years), **b** Clinical Frailty Scale (1–3; 4; >  = 5) and **c** PaO_2_/FiO_2_ ratio at Day-1 of ICU admission

Mortality was 41%, 45%, and 46% at Day-28, Day-60, and Day-90, respectively (Additional file 1: Table S1). Mortality at Day-90 increased with the age and the Clinical Frailty Scale (Fig. 1). Indeed, Day-90 mortality increased from 39% in the patients between 70 and 74 years to 47% and 67% in the groups of patients between 75 and 79 years and those over 80 years old, respectively (*p* < 0.001) (Fig. 2a). Similarly, mortality at Day-90 was 40%, 61%, and 71% in the patients' groups with Clinical Frailty Scale from 1–3; 4; and ≥ 5, respectively (*p* < 0.001) (Fig. 2b). The mortality was also higher in patients intubated during their ICU stay ranging from 44 to 74% (Additional file 1: Figure S2). Of note, during the period of the first 14 days following the ICU admission, 253/1,199 (21%) of the patients had a life-sustaining treatment limitation decision, whom 223 (88%) died at day 90 (207 (82%) while in the ICU).

### Predictive factors of mortality at Day-90

Results of the multivariable analysis are reported in Table 2. Because of multicollinearity observed between age and Clinical Frailty Scale, invasive mechanical ventilation at Day-1 and PaO_2_/FiO_2_ ratio, renal replacement therapy and the renal component of the SOFA, only Clinical Frailty Scale, PaO_2_/FiO_2_ ratio, and the renal component of the SOFA were retained in the model. Day-1 patients’ characteristics significantly associated with a higher 90-Day mortality rate identified by the Cox regression model after center stratification were older age, diabetes, higher cardiovascular component of the SOFA score, lower PaO_2_/FiO_2,_ and a shorter time between first symptoms and ICU admission (Table 2). The same analysis re-run of missing after multiple imputations data (Additional file 1: Table S2) yielded similar conclusions. Interestingly, being admitted to the ICU after March 29 was also associated with a better outcome (Additional file 1: Figure S3). Kaplan–Meier survival estimates according to age categories, Clinical Frailty Scale, and PaO_2_/FiO_2_ ratio at Day-1 of ICU admission are provided in Fig. 2.

**Table 2:** Predictive patient factors associated with Day-90 mortality in critically ill patients older than 70 years old with COVID-19 stratified on the center variable

	No.	Univariate HR (95% CI)	*P *value	Multivariate HR (95% CI)	*P *value
Age, years	1199	–	< 0.001	–	
70–75		–		–	
75–79		1.32 (1.08–1.60)		–	
80–84		2.09 (1.64–2.68)		–	
85–91		4.09 (2.97–5.65)		–	
Clinical Frailty Scale	1085	–	< 0.001	–	< 0.001
1–3		–		–	
4		2.14 (1.71–2.68)		2.24 (1.63–3.09)	
5–9		2.81 (2.17–3.64)		2.83 (1.96–4.08)	
Body mass index, kg/m^2^	1096		0.435	–	0.103
< 25				–	
25–29		0.96 (0.77–1.20)		1.10 (0.83–1.48)	
30–34		0.85 (0.64–1.11)		0.78 (0.55–1.12)	
35–39		0.89 (0.61–1.31)		0.90 (0.53–1.51)	
≥ 40		1.33 (0.83–2.13)		1.26 (0.72–2.22)	
Diabetes	1184	1.43 (1.20–1.71)	< 0.001	1.42 (1.10–1.82)	0.043
Hypertension	1189	1.03 (0.87–1.23)	0.726	0.87 (0.68–1.12)	0.697
Immunodepression	1186	1.31 (0.99–1.74)	0.066	0.97 (0.63–1.48)	0.298
Time between first signs and ICU admission	1109		< 0.001		0.003
< 4 days		–		–	
4–7 days		0.88 (0.70–1.12)		0.87 (0.63–1.18)	
≥ 8 days		0.50 (0.40–0.64)		0.61 (0.44–0.84)	
SOFA Cardiovascular component ≥3	1160	1.74 (1.47–2.07)	< 0.001	2.13 (1.66–2.74)	< 0.001
SOFA renal component ≥3	1140	1.84 (1.37–2.49)	< 0.001	1.39 (0.94–2.05)	0.909
Invasive mechanical ventilation at Day-1	1199	1.66 (1.38–1.99)	< 0.001	–	
Renal replacement therapy at Day-1	1188	2.50 (1.67–3.73)	< 0.001	–	
ICU admission after March 29^th^	1199	0.67 (0.56–0.80)	< 0.001	0.70 (0.55–0.89)	< 0.001
PaO_2_/FiO_2_ ratio	868		< 0.001	–	0.001
200 < PaO_2_/FiO_2_		–		–	
100 < PaO_2_/FiO_2_ ≤ 200		1.14 (0.90–1.44)		1.28 (0.97–1.69)	
PaO_2_/FiO_2_ ≤ 100		1.68 (1.30–2.16)		2.35 (1.73–3.19)	

Age, invasive mechanical ventilation and renal replacement therapy variables were excluded from multivariate analysis for multicollinearity issue

*CI* confidence interval, *HR* hazard ratio, *ICU* intensive care unit, *SOFA* Sequential Organ Failure Assessment

### Propensity score analysis

**Fig. 3:**
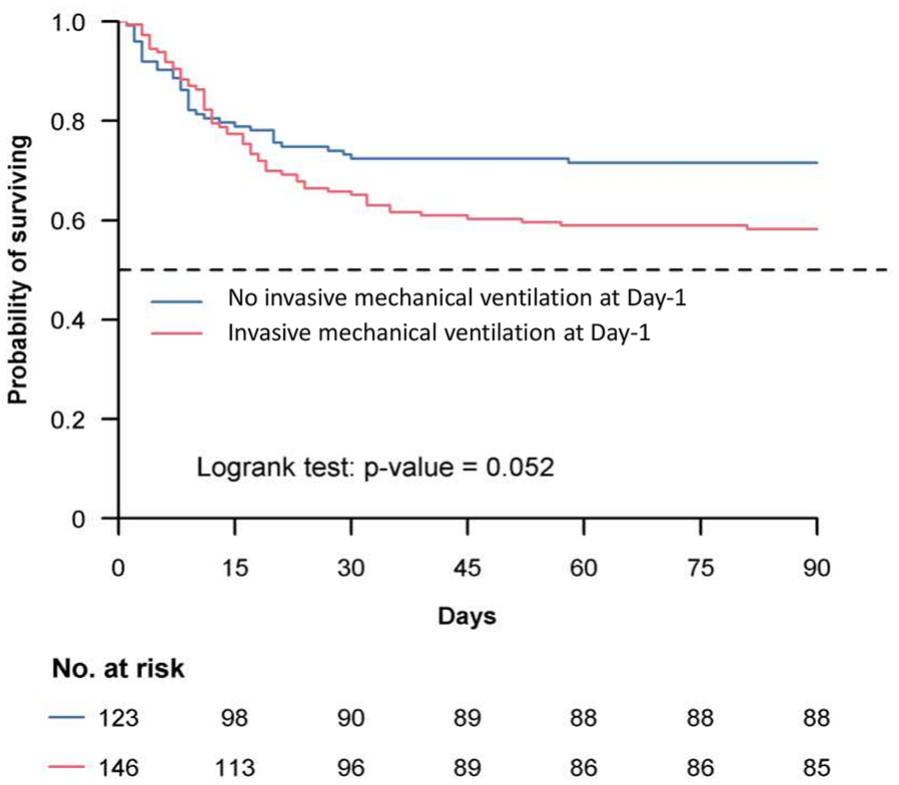
Kaplan–Meier survival estimates during the 90 days following ICU admission in propensity score-matched patients

Six hundred and forty-four patients had a cardiovascular component of the SOFA < 2, comprising 425 patients intubated on Day-1 and 219 initially treated without invasive mechanical ventilation. These two groups differed in several respects (Additional file 1: Table S3). Patients intubated on Day-1 had a higher SOFA cardiovascular component and were more likely admitted to the ICU before March 28. Interestingly, their Clinical Frailty Scale, their BMI, the time between first symptoms and ICU admission, and the PaO_2_/FiO_2_ ratio were not different. After weighting on the Inverse Probability Weighting Treatment using propensity score estimated in 269 patients with no missing values, 123 non-intubated patients were compared to 146 patients intubated at Day-1 with a similar medical history and initial severity Additional file 1: Table S3). We found a significantly different Day-90 mortality (28% in the non-intubated group vs. 42% in the intubated group; HR 1.68; 95% CI 1.24–2.27; *p* < 0.001) (Fig. 3). A similar analysis performed after multiple imputations of missing data (i.e., 644 patients) yielded similar conclusions (HR 1.33; 95% CI 1.11–1.59; *p* = 0.002).

## Discussion

Herein, we report the characteristics, management, and outcomes of a large prospective cohort of old critically ill patients during the first wave of the COVID-19 outbreak. Patients over 70 years represented 28% of the COVID-19 population admitted during that period of 8 weeks in the participating ICUs. Their overall Day-90 mortality was 46%, which increased with the age and the Clinical Frailty Scale and reached 67% for the patients over 80 years. Older age, diabetes, a longer time between first symptoms and ICU admission, a SOFA cardiovascular component ≥ 3, a lower PaO_2_/FiO_2_ ratio, and being admitted to the ICU during the first month of the pandemic were independent risk factors of Day-90 mortality. Noticeably, our propensity score analysis suggests that an early invasive mechanical ventilation strategy seemed associated with a worse prognosis in that population.

The mortality of elderly patients admitted in the ICU for SARS-Cov-2-related ARDS varied from 77 to 84% [1]. These mortality rates appear very high compared to those reported in ARDS outside COVID-19 [12, 23]. For instance, the Large Observational Study to Understand the Global Impact of Severe Acute Respiratory Failure (LUNG SAFE) reported Day-90 mortality rates of 47%, 51%, and 50% for the 70–74 years, 75–79, and > 80 years old patients, respectively (unpublished data, personal communication from the authors) [24]. Our Day-90 mortality (46%) contrasts with early reports (1–3) and the large German cohort of 10,021 patients (923 patients over 70 years) [4] despite a large proportion of patients intubated during their ICU stay in our study (78%). It was, however much higher than the 25% Day-90 mortality observed in the rest of the population of the COVID-ICU cohort (i.e., patients < 70 years old) [15]. Besides, the mortality of our patients over 80 years old seems higher when compared with same-age patients with non-COVID-19-related ARDS, planned [25], or unplanned ICU admission [26]. Several factors such as triage policy before ICU admission, ICU resources at the time of the pandemic, ICU case volume [27] and patients’ comorbidities may explain these discrepancies.

Before the context of COVID-19, frailty as measured with the Clinical Frailty Scale in elderly critically ill patients was strongly associated with Day-30 mortality [26]. This tool was even a better predictor of mortality than SOFA score [25] or classical geriatric scales [26]. Recently, in a large observational study performed in the United Kingdom that enrolled 1564 COVID-19 patients with a median age of 74 years, and more than 50% of the population with a Clinical Frailty Scale > 4, the crude hazard ratio (95% confidence interval) for mortality were 3.12 (2.05–4.76) and 4.41 (2.90–6.71) for those with a Clinical Frailty Scale of 5–6 and 7 to 9, respectively [11]. However, the overall low Clinical Frailty Scale reported in our study and our low proportion of vulnerable or frail patients suggest that a significant triage was performed before ICU admission [28]. No national ICU admission criteria policy was provided at the time of the study, and the ICU admission decision was left to the discretion of the physicians in charge of the patient. Whether this triage resulted from intensivist’s evaluation, non-intensivists practitioner’s judgment, ICU beds occupancy, or the patients themselves should be further investigated.

Old patients admitted to the ICU with COVID-19 are at increased risk of death [3, 29] and the decision of ICU admission can be challenging [8]. The use of the Clinical Frailty Scale has proven to be helpful in this context [9]. Besides, the respect of the patient’s wishes and values, expressed directly by the patient via advance directives or reported by the healthcare surrogate should have to be taken into consideration [30]. In old patients with an uncertain prognosis, it can be particularly difficult to decide whether or not to admit to the ICU and provide invasive treatments such as mechanical ventilation. In such circumstances, an “ICU-trial of limited-time” has been proposed [31]. However, in the context of COVID-19, this strategy could be challenging as a long invasive mechanical duration is often required to see any clinical improvement. In other words, an ICU trial with a too-short limited-time could lead to misinterpretation and ethical misconduct. This important point is reinforced by the extremely long durations of invasive mechanical ventilation, and ICU length of stay observed in our surviving patients.

Beyond the admission of elderly patients in the ICU, the decision of the timing of intubation remains crucial. The majority of our patients (62%) were intubated on ICU Day-1. Interestingly, apart from obvious reasons such as hemodynamic instability, relevant clinical differences were scarce between patients who were intubated upon admission and those who were not. For instance, their Clinical Frailty Scale, time between first symptoms and ICU admission, and PaO_2_/FiO_2_ ratio were not significantly different, suggesting that the decision of intubation on admission was mainly driven by the experience of the physicians and the limited knowledge of this new disease at that time. As reported by others [32], the proportion of patients being intubated upon ICU admission during the first period of the study decreased from 67 to 56% during the last month (after March 29th, 2020), with being admitted in that latter period independently associated with a lower Day-90 mortality. An early intubation strategy was even associated with a poorer outcome in our matching analysis while further studies are warranted to confirm this finding. Less reluctance of the caregivers to provide non-invasive oxygen strategies along the first COVID-19 wave has been reported [15], but the benefit in terms of survival is still uncertain [33]. These strategies seem promising in that at-risk population where patients receiving invasive mechanical ventilation are more likely to experience long-term physical, neuropsychiatric, and quality of life impairments [34, 35].

Our study is a large international cohort of old critically ill patients with detailed characteristics and Day-90 outcome. However, despite a large number of participating ICUs, our population sample may be prone to selection biases that may limit generalizability. Since the study was mainly conducted in France (1115, 41 and 43 patients in France, Switzerland, and Belgium, respectively) during a period with high pressure on the health system and before the publication of several core randomized trials [36, 37], our findings may differ during subsequent COVID-19 outbreaks, and in countries with different public health care organizations, ICU admission policy, or ICU resources [4]. Comparison with further studies from other countries will help to better allocate health care resources and determine the indications and contra-indications of non-invasive ventilatory strategies in this specific population. Besides, we only provided data on patients who were admitted to the ICU, and no information was available on treatments before ICU admission nor on patients for whom an ICU admission was denied in the participating ICUs. Besides, important detailed information is also lacking regarding therapy limitations. This information would have allowed a thorough investigation of ICU-admission criteria used during this surge of ICU resources.

## Conclusions

During the first COVID-19 pandemic wave, patients over 70 years old represented more than a quarter of the COVID-19 population in the participating ICUs of that study. Their overall Day-90 mortality was 46% with a dismal prognosis in patients older than 80 years old. Given the very long duration of mechanical ventilation as well as a prolonged ICU and hospital stay in the survivors, further studies are urgently warranted to evaluate the long-term psychological, neurocognitive, and functional outcomes of this high-risk and vulnerable population.

## Supplementary Information

**Additional file 1.** Detailed description of the data collection and statistal analysis and complementary tables and figures

## Data Availability

The datasets used and/or analyzed during the current study are available from the corresponding author on reasonable request.
