# Supplementary material for: Characteristics, management, and prognosis of elderly patients with COVID-19 admitted in the ICU during the first wave: insights from the COVID-ICU study: Prognosis of COVID-19 elderly critically ill patients in the ICU
Source: Ann Intensive Care. 2021 May 14;11:77. doi: 10.1186/s13613-021-00861-1 (PMC8120254; doi:10.1186/s13613-021-00861-1)
Supplement: Supplementary file 1 — Additional file 1. Detailed description of the data collection and statistal analysis and complementary tables and figures [file 13613_2021_861_MOESM1_ESM.docx]

**Characteristics, management, and prognosis of elderly patients with COVID-19 admitted in the ICU during the first wave**

***Insights from the Covid-ICU study***

**Additional Files**

Martin Dres^1,2^, David Hajage^3,4^, Said Lebbah^4^, Antoine Kimmoun^5,6^, Tai Pham^7,8^, Gaetan Beduneau^9,10^, Alain Combes^11,12^, Alain Mercat^13^, Bertrand Guidet^14^, Alexandre Demoule^1,2^, Matthieu Schmidt^11,12^ and the COVID-ICU investigators

^1^ AP-HP, Groupe Hospitalier Universitaire APHP-Sorbonne Université, site Pitié-Salpêtrière, Service de Pneumologie, Médecine intensive Réanimation (Département R3S), Paris, France

^2^ Sorbonne Université, INSERM, UMRS_1158 Neurophysiologie respiratoire expérimentale et clinique, Paris, France

^3^ Sorbonne Université, INSERM, Institut Pierre Louis d’Epidémiologie et de Santé Publique

^4^ AP-HP, Département de Santé Publique, Centre de Pharmacoépidémiologie, Paris, France

^5^ Université de Lorraine, CHRU de Nancy, Service de Médecine Intensive et Réanimation Brabois

^6^ INSERM U1116, Vandoeuvre-les-Nancy, France

^7^ Service de Médecine Intensive – Réanimation, Hôpital Bicêtre, Hôpitaux universitaires Paris-Saclay, Le Kremlin-Bicêtre, France

^8^ Équipe d’Épidémiologie Respiratoire Intégrative, Center for Epidemiology and Population Health (CESP), Université Paris-Saclay, UVSQ, Univ. Paris-Sud, Inserm, Villejuif, France

^9^ Normandie Univ, UNIROUEN, EA 3830

^10^ Rouen University Hospital, Medical Intensive care Unit, F-76000 Rouen, France

^11^ Sorbonne Université, Institute of Cardiometabolism and Nutrition

^12^ APHP, Pitié–Salpêtrière Hospital, Medical Intensive Care Unit, Paris, France

^13^ Département de Médecine Intensive-Réanimation et Médecine Hyperbare, Centre Hospitalier Universitaire d'Angers, Université d'Angers, France

**Data collection**

Day-1 was defined as the first day when the patient was in ICU at 10 am. Each day, the study investigators completed a standardized electronic case report form. Baseline information collected at ICU admission were: age, sex, body mass index (BMI), active smoking, Simplified Acute Physiology Score (SAPS) II score [1], Sequential Organ Failure Assessment (SOFA) [2], comorbidities, immunodeficiency (if present), Clinical Frailty Scale [3], the date of the first symptom, and dates of the hospital and ICU admissions. The Clinical Frailty Scale is an ordinal hierarchical scale of 9 ranks, with a score of 1 being very fit, 2 well, 3 managing well, 4 vulnerable, 5 mildly frail, 6 moderately frail, 7 severely frail, 8 very severely frail, and 9 terminally ill. Eventually, patients were categorized into three Clinical Frailty Score groups: 1 to 3 (fit), 4 (vulnerable, but not frail), and 5 to 9 (frail) for our analyses. Immunodeficiency was defined as hematological malignancies, an active solid tumor or having received specific anti-tumor treatment within a year, solid-organ transplant, human immunodeficiency virus, or immunosuppressants, whereas chronic respiratory disease including asthma, chronic obstructive pulmonary disease, restrictive pulmonary disease, or apnea. Mode of respiratory support (invasive mechanical ventilation, non-invasive mechanical ventilation, oxygen mask, high flow nasal oxygen), the fraction of inspired oxygen (FiO_2_), the arterial partial pressure of oxygen (PaO_2_), PaO_2_/FiO_2_ ratio, the use of neuromuscular blockades, corticosteroids (regardless of the indication and the dose) were collected. For patients who received standard oxygen therapy, their Day-1 PaO_2_/FiO_2_ was calculated by converting O_2_ flow to estimated FiO_2_ [4]. We also collected complications and organ dysfunction over the ICU stay including acute renal failure requiring renal replacement therapy, thromboembolic complications (distal venous thrombosis or proven pulmonary embolism by either pulmonary CT angiography or cardiac echography), ventilator-associated pneumonia, and cardiac arrest. Ventilator-associated pneumonia was defined by either a bronchoalveolar lavage cultures growing ≥10^4^cfu/mL, blind protected specimen brush distal growing ≥10^3^cfu/mL, or endotracheal aspirates growing ≥10^6^cfu/mL. Patient outcomes included duration of mechanical ventilation, vital status at ICU and hospital discharge, and 28, 60, and 90 days after ICU admission. Lastly, life-sustaining treatment decisions were also collected.

**Statistical Analyses**

Characteristics of patients were described as frequencies and percentages for categorical variables, whereas continuous variables were reported as mean and standard deviation or median and interquartile range. Categorical variables were compared by chi-square or Fisher's exact test, and continuous variables were compared by Student's t-test or Wilcoxon's rank-sum test. Kaplan-Meier overall survival curves until Day-90 were computed, and were compared using log-rank tests.

Baseline risk factors of death at Day-90 were assessed using univariate and multivariate Cox regression model stratified on the center variable. Baseline variables (i.e., obtained during the first 24 h in the ICU) included in the multivariate model were defined a priori, and no variable selection was planned. These variables were: age, Clinical Frailty Scale, BMI, diabetes, treated hypertension, immunodepression, the time between first signs and ICU admission, ICU admission period, SOFA cardiovascular system component, SOFA renal component, and PaO_2_/FiO_2_ ratio, with a sensitivity analysis using a Cox model stratified on the centre variable. Proportional hazard assumption was assessed by inspecting the scaled Shoenfeld residuals and Harrel’s test [5]. Multicollinearity was assessed using the variance inflation factor. Multiple imputations were used to replace missing values when appropriate. Ten copies of the dataset were created with the missing values replaced by imputed values, based on observed data including outcomes and baseline characteristics of participants. Each dataset was then analyzed and the results from each dataset were pooled into a final result using Rubin’s rule [6]. Hazard ratios and their 95% confidence interval were estimated.

To assess invasive mechanical ventilation effect on Day-90 mortality, we used a Cox proportional hazard model weighted on inverse probability of treatment weighting (IPTW) using propensity score (PS) defined as the predictive probability of invasive mechanical ventilation conditional on measured baseline covariates [7]. The population included for propensity score analysis included only the patients with a cardiovascular component of the SOFA score < 2. Indeed, patients with a cardiovascular component of the SOFA score of 2 or more were almost systematically invasively ventilated and therefore could not be included for propensity score analysis (positivity issue). A multivariate logistic regression model was performed to estimate the PS for each patient in that population. The variables included in the PS estimating model were: age, clinical frailty scale, chronic respiratory disease, BMI, time between first symptoms and ICU admission, ICU admission period, the cardiovascular component of the SOFA, the renal component of the SOFA, and PaO_2_/FiO_2_ ratio. To assess the balance of measured covariates between treatment groups, we used the standardized mean differences before and after PS weighting [8]. For each covariate, a standard difference less than 0.1 was considered as negligible [7]. Then, a Cox proportional hazard model weighted on IPTW was performed to estimate the average treatment effect in the entire eligible population [7]. Hazard ratio and its 95% confidence interval were then estimated for the Day-90 mortality associated with invasive mechanical ventilation at Day-1. This analysis was performed on the complete cases data set, and a sensitivity analysis was performed using multiple imputations due to missing data.

All analyses were performed at a two-sided α level of 5% and conducted with R version 3.5.1 (R Foundation for Statistical Computing, Vienna, Austria).

**References**

1. Le Gall JR, Lemeshow S, Saulnier F. A new Simplified Acute Physiology Score (SAPS II) based on a European/North American multicenter study. JAMA. 1993;270:2957–63.

2. Vincent JL, Moreno R, Takala J, Willatts S, De Mendonça A, Bruining H, et al. The SOFA (Sepsis-related Organ Failure Assessment) score to describe organ dysfunction/failure. On behalf of the Working Group on Sepsis-Related Problems of the European Society of Intensive Care Medicine. Intensive Care Med. 1996;22:707–10.

3. Juma S, Taabazuing M-M, Montero-Odasso M. Clinical Frailty Scale in an Acute Medicine Unit: a Simple Tool That Predicts Length of Stay. Can Geriatr J. 2016;19:34–9.

4. Vincent J-L, Rello J, Marshall J, Silva E, Anzueto A, Martin CD, et al. International study of the prevalence and outcomes of infection in intensive care units. JAMA. 2009;302:2323–9.

5. Grambsch P, Therneau T. Proportional hazards tests and diagnostics based on weighted residuals. Biometrika. 1994;81:515–26.

6. Sterne JAC, White IR, Carlin JB, Spratt M, Royston P, Kenward MG, et al. Multiple imputation for missing data in epidemiological and clinical research: potential and pitfalls. BMJ. 2009;338:b2393–b2393.

7. Austin PC. An Introduction to Propensity Score Methods for Reducing the Effects of Confounding in Observational Studies. Multivariate Behavioral Research. 2011;46:399–424.

8. Austin PC. Balance diagnostics for comparing the distribution of baseline covariates between treatment groups in propensity-score matched samples. Statistics in Medicine. 2009;28:3083–107.

**Figure S1: Flow chart of the study**

*ICU, intensive care unit*

**Figure S2: Day-90 mortality in intubated patients according to age categories and timing of intubation**

**Figure S3: Kaplan–Meier survival estimates during the 90 days following ICU admission, according to period of ICU admission, before March 28^th^ 2020 or after March 29^th^ 2021.**


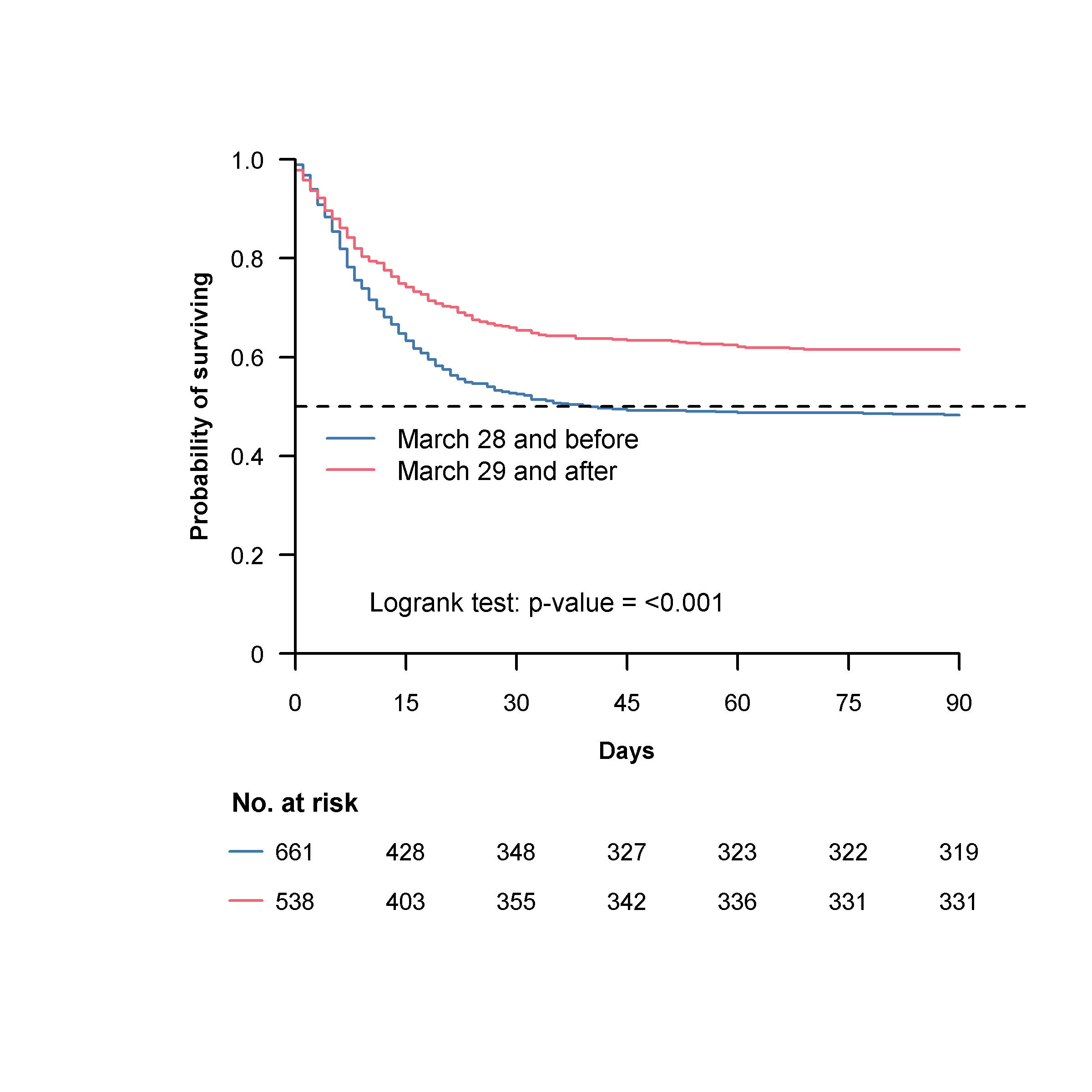


**Table S1: Major complications and Outcomes of patients according to their intubation status in the ICU.**

*ICU, intensive care unit; LOS, length of stay*

|  |  | **Invasive Mechanical Ventilation** | |  |
| --- | --- | --- | --- | --- |
|  | **All patients**  **(n=1,199)** | **No**  **(n=261)** | **Yes**  **(n=938)** | ***P* value** |
| Cardiac arrest | 59 (5) | 1 (0) | 58 (6) | <0.001 |
| Pulmonary embolism | 83 (7) | 11 (4) | 72 (8) | 0.049 |
| Proved distal venous thrombosis | 68 (6) | 3 (1) | 65 (7) | <0.001 |
| Ventilator associated pneumonia | 487 (45) | 24 (12) | 463 (53) | <0.001 |
| Bacterial coinfection | 52 (5) | 10 (5) | 42 (5) | 0.792 |
| Secondary bacterial infection | 613 (66) | 274 (60) | 339 (72) | <0.001 |
| Duration of invasive ventilation, days | 7 (0-14) | - | 10 (6-17) | - |
| Duration of invasive ventilation in surviving patients, days | 6 (0-14) | - | 12 (8-18) | - |
| ICU LOS, days | 12 (5-23) | 4 (2-8) | 16 (8-28) | <0.001 |
| ICU LOS in surviving patients, days | 16 (7-29) | 5 (3-9) | 22 (13-38) | <0.001 |
| ICU mortality | 493 (41) | 48 (18) | 445 (48) | <0.001 |
| Still in ICU at Day 28 | 236 (20) | 3 (1) | 233 (25) | <0.001 |
| Hospital LOS, days | 19 (10-33) | 15 (9-24) | 21 (10-37) | <0.001 |
| Hospital LOS in surviving patients, days in surviving patients, days | 27 (17-46) | 17 (12-26) | 33 (22-57) | <0.001 |
| Hospital mortality | 535 (47) | 72 (28) | 463 (53) | <0.001 |
| Day-28 mortality | 490 (41) | 73 (28) | 417 (45) | <0.001 |
| Day-60 mortality | 541 (45) | 73 (28) | 468 (50) | <0.001 |
| Day-90 mortality | 549 (46) | 73 (28) | 476 (51) | <0.001 |

Values are expressed as median (interquartile range) or n (%).

**Table S2: Predictive Patient Factors Associated with 90-day mortality in 1,199 elderly critically ill adults with COVID-19 stratified on the centre variable and with multiple imputations**

| Variables | Hazard Ratio  (95% CI) | P value |
| --- | --- | --- |
| Clinical Frailty Scale |  | <0.001 |
| 1 – 3 | reference |  |
| 4 | 1.84 (1.39 - 2.42) |  |
| 5 – 9 | 2.62 (1.91 - 3.59) |  |
| Body mass index, kg/m2 |  | 0.059 |
| < 25 | reference |  |
| 25 – 29 | 1.12 (0.87 - 1.43) |  |
| 30 – 34 | 0.78 (0.57 - 1.08) |  |
| 35 – 39 | 0.71 (0.46 - 1.09) |  |
| ≥40 | 1.11 (0.64 - 1.93) |  |
| Diabetes | 1.20 (0.97 - 1.48) | 0.098 |
| Hypertension | 0.93 (0.76 - 1.15) | 0.517 |
| Immunocompromised status | 1.08 (0.78 - 1.51) | 0.647 |
| Time between first signs and ICU admission |  | <0.001 |
| < 4 days | reference |  |
| 4 – 7 days | 0.93 (0.70 - 1.24) |  |
| ≥ 8 days | 0.60 (0.45 - 0.82) |  |
| SOFA Cardiovascular component ≥3 | 1.82 (1.48 - 2.24) | <0.001 |
| SOFA Renal Component ≥3 | 1.26 (0.87 - 1.82) | 0.227 |
| ICU admission after March 29th | 0.68 (0.56 - 0.84) | <0.001 |
| PaO2/FiO2 ratio |  | 0.002 |
| > 200 | reference |  |
| 101 – 200 | 1.13 (0.87 - 1.47) |  |
| ≤ 100 | 1.65 (1.24 - 2.21) |  |

*CI, confidence interval; SOFA: sequential organ failure assessment; ICU: intensive care unit; FiO_2_: fraction of inspired oxygen; PaO_2_: partial pressure of oxygen.*

**Table S3: Demographic Characteristics and 90-Day mortality according to their ICU day-1 Intubation Status in a propensity score population analysis**

|  | | |  | | | **Baseline population**  **(n = 644)** | | |  | | | **Complete case population (n = 269)** | | |  | | | **Before imputation**  **(n = 269)** | | | **After imputation**  **(n = 644)** | |
| --- | --- | --- | --- | --- | --- | --- | --- | --- | --- | --- | --- | --- | --- | --- | --- | --- | --- | --- | --- | --- | --- | --- |
| **Variable** | **Label** | | | **No intubation**  **(n = 425)** | | **Intubation**  **(n = 219)** | | **p** | | | **No intubation**  **(n = 123)** | **Intubation**  **(n = 146)** | **p** | | | **SMD**  **observed** | | **SMD**  **weighted** | | **SMD**  **observed** | | **SMD**  **weighted** |
| **Age, years** |  | | |  | |  | | 0.01 | | |  |  | 0.31 | | |  | |  | |  | |  |
|  | 70 – 74 | | | 212 (50) | | 132 (60) | |  | | | 67 (54) | 91 (62) |  | | | 0.159 | | 0.079 | | 0.210 | | 0.014 |
|  | 75 – 79 | | | 131 (31) | | 63 (29) | |  | | | 39 (32) | 42 (29) |  | | | 0.064 | | 0.083 | | 0.045 | | 0.009 |
|  | ≥ 80 | | | 82 (19) | | 24 (11) | |  | | | 17 (14) | 13 (9) |  | | | 0.155 | | 0.003 | | 0.234 | | 0.023 |
| **Clinical frailty scale** |  | | |  | |  | | 0.08 | | |  |  | 0.35 | | |  | |  | |  | |  |
|  | 1-3 | | | 289 (74) | | 157 (82) | |  | | | 97 (79) | 125 (86) |  | | | 0.177 | | 0.015 | | 0.179 | | 0.044 |
|  | 4 | | | 60 (15) | | 21 (11) | |  | | | 15 (12) | 12 (8) |  | | | 0.131 | | 0.014 | | 0.121 | | 0.014 |
|  | 5-9 | | | 42 (11) | | 13 (7) | |  | | | 11 (9) | 9 (6) |  | | | 0.105 | | 0.034 | | 0.117 | | 0.070 |
| **Chronic respiratory disease** |  | | | 103 (25) | | 49 (23) | | 0.62 | | | 33 (27) | 32 (22) | 0.35 | | | 0.114 | | 0.014 | | 0.045 | | 0.030 |
| **SOFA Cardiovascular system ^a^** |  | | |  | |  | | <0.001 | | |  |  | <0.001 | | |  | |  | |  | |  |
|  | 0 | | | 352 (83) | | 139 (63) | |  | | | 106 (86) | 89 (61) |  | | | 0.595 | | 0.021 | | 0.447 | | 0.006 |
|  | 1 | | | 73 (17) | | 80 (37) | |  | | | 17 (14) | 57 (39) |  | | | 0.595 | | 0.021 | | 0.447 | | 0.006 |
| **SOFA Renal** |  | | |  | |  | | 0.21 | | |  |  | 0.75 | | |  | |  | |  | |  |
|  | 0 | | | 304 (75) | | 154 (74) | |  | | | 91 (74) | 107 (73) |  | | | 0.016 | | 0.055 | | 0.036 | | 0.020 |
|  | 1 | | | 52 (13) | | 34 (16) | |  | | | 17 (14) | 24 (16) |  | | | 0.073 | | 0.079 | | 0.097 | | 0.010 |
|  | 2 | | | 25 (6) | | 8 (4) | |  | | | 8 (7) | 6 (4) |  | | | 0.107 | | 0.019 | | 0.113 | | 0.009 |
|  | 3 | | | 7 (2) | | 8 (4) | |  | | | 3 (2) | 6 (4) |  | | | 0.094 | | 0.023 | | 0.120 | | 0.042 |
|  | 4 | | | 15 (4) | | 5 (2) | |  | | | 4 (3) | 3 (2) |  | | | 0.074 | | 0.021 | | 0.104 | | 0.014 |
| **Body mass index, kg/m^2^** |  | | |  | |  | | 0.23 | | |  |  | 0.36 | | |  | |  | |  | |  |
|  | < 25 | | | 98 (25) | | 35 (18) | |  | | | 31 (25) | 23 (16) |  | | | 0.235 | | 0.005 | | 0.195 | | 0.011 |
|  | 25 – 29 | | | 188 (49) | | 101 (51) | |  | | | 59 (48) | 81 (55) |  | | | 0.150 | | 0.009 | | 0.047 | | 0.014 |
|  | 30 – 34 | | | 64 (17) | | 41 (21) | |  | | | 20 (16) | 28 (19) |  | | | 0.076 | | 0.011 | | 0.108 | | 0.010 |
|  | 35 – 39 | | | 25 (6) | | 17 (9) | |  | | | 10 (8) | 10 (7) |  | | | 0.048 | | 0.010 | | 0.064 | | 0.014 |
|  | ≥ 40 | | | 11 (3) | | 5 (3) | |  | | | 3 (2) | 4 (3) |  | | | 0.019 | | 0.002 | | 0.021 | | 0.006 |
| **Time between first signs and ICU admission** |  | | |  | |  | | 0.99 | | |  |  | 0.69 | | |  | |  | |  | |  |
|  | < 4 | | | 66 (16) | | 32 (16) | |  | | | 21 (17) | 26 (18) |  | | | 0.019 | | 0.021 | | 0.011 | | 0.014 |
|  | 4 – 7 | | | 133 (33) | | 67 (33) | |  | | | 50 (41) | 52 (36) |  | | | 0.103 | | 0.023 | | 0.014 | | 0.019 |
|  | ≥ 8 | | | 205 (51) | | 102 (51) | |  | | | 52 (42) | 68 (47) |  | | | 0.086 | | 0.007 | | 0.012 | | 0.009 |
| **ICU admission period** |  | | |  | |  | | 0.007 | | |  |  | 0.93 | | |  | |  | |  | |  |
|  | - March 28 | | | 201 (47) | | 128 (58) | |  | | | 71 (58) | 85 (58) |  | | | 0.010 | | 0.019 | | 0.224 | | 0.035 |
|  | March 29 - | | | 224 (53) | | 91 (42) | |  | | | 52 (42) | 61 (42) |  | | | 0.010 | | 0.019 | | 0.224 | | 0.035 |
| **PaO_2_/FiO_2_** |  | | |  | |  | | 0.55 | | |  |  | 0.78 | | |  | |  | |  | |  |
|  | > 300 | | | 12 (8) | | 18 (9) | |  | | | 9 (7) | 13 (9) |  | | | 0.058 | | 0.021 | | 0.095 | | 0.021 |
|  | 201 - 300 | | | 30 (19) | | 50 (25) | |  | | | 23 (19) | 33 (23) |  | | | 0.096 | | 0.035 | | 0.080 | | 0.012 |
|  | 101 - 200 | | | 65 (42) | | 83 (41) | |  | | | 56 (46) | 59 (40) |  | | | 0.103 | | 0.021 | | 0.181 | | 0.023 |
|  | ≤ 100 | | | 47 (31) | | 51 (25) | |  | | | 35 (28) | 41 (28) |  | | | 0.008 | | 0.043 | | 0.193 | | 0.019 |
| **Day- 90 mortality** |  | | | 148 (35) | | 93 (42) | | 0.06 | | | 35 (28) | 61 (42) | 0.02 | | | - | | - | | - | | - |
| **HR (CI 95, p)** | | - | | | - | | - | | | - | | - | | - | | | - | | 1.68 (1.24 – 2.27,  p < 0.001) | | | 1.33 (1.11 – 1.59,  p = 0.002) |

*Values are expressed as n (%).*

*ICU, intensive care unit; SOFA, Sequential Organ Failure Assessment; SMD, Standardized Mean Difference, SMD weighted: SMD weighted on Inverse Probability Weighting Treatment (IPWT) using propensity score estimated*

*^a^ Only patients with a SOFA cardiovascular component < 2 were included in the PS analysis*
